# Locality Constrained Joint Dynamic Sparse Representation for Local Matching Based Face Recognition

**DOI:** 10.1371/journal.pone.0113198

**Published:** 2014-11-24

**Authors:** Jianzhong Wang, Yugen Yi, Wei Zhou, Yanjiao Shi, Miao Qi, Ming Zhang, Baoxue Zhang, Jun Kong

**Affiliations:** 1 College of Computer Science and Information Technology, Northeast Normal University, Changchun, China; 2 School of Mathematics and Statistics, Northeast Normal University, Changchun, China; 3 National Engineering Laboratory for Druggable Gene and Protein Screening, Northeast Normal University, Changchun, China; 4 Key Laboratory of Intelligent Information Processing of Jilin Universities, Northeast Normal University, Changchun, China; 5 School of Statistics, Capital University of Economics and Business, Beijing, China; Universidad de Castilla-La Mancha, Spain

## Abstract

Recently, Sparse Representation-based Classification (SRC) has attracted a lot of attention for its applications to various tasks, especially in biometric techniques such as face recognition. However, factors such as lighting, expression, pose and disguise variations in face images will decrease the performances of SRC and most other face recognition techniques. In order to overcome these limitations, we propose a robust face recognition method named Locality Constrained Joint Dynamic Sparse Representation-based Classification (LCJDSRC) in this paper. In our method, a face image is first partitioned into several smaller sub-images. Then, these sub-images are sparsely represented using the proposed locality constrained joint dynamic sparse representation algorithm. Finally, the representation results for all sub-images are aggregated to obtain the final recognition result. Compared with other algorithms which process each sub-image of a face image independently, the proposed algorithm regards the local matching-based face recognition as a multi-task learning problem. Thus, the latent relationships among the sub-images from the same face image are taken into account. Meanwhile, the locality information of the data is also considered in our algorithm. We evaluate our algorithm by comparing it with other state-of-the-art approaches. Extensive experiments on four benchmark face databases (ORL, Extended YaleB, AR and LFW) demonstrate the effectiveness of LCJDSRC.

## Introduction

In the past two decades, face recognition has become one of the most active and challenging research topics in pattern recognition and computer vision fields due to its wide range of applications in biometrics, human-computer interaction, information security and so on [1], [2]. Although many researchers have proposed various algorithms for face recognition [1], [2], it is still a challenging problem [3], [4]. This is because the appearance of real-world face images is always affected by illumination condition, aging, pose, facial expression and disguise variances. Moreover, some other problematic factors such as occlusion and noise will also impair the performances of face recognition algorithms.

Recently, sparse representation (or sparse coding) techniques have drawn wide interest and been successfully used in signal, image, video processing and biometric applications [5]. Motivated by sparse representation, a novel face recognition method named Sparse Representation-based Classification (SRC) [6] was proposed by Wright et al. In SRC, a query image is first sparsely linear coded by the original training images, and then the classification is performed by checking which class leads to the minimal representation residual of the query image. Since the experimental results in Wright et al.'s pioneer work showed that the SRC achieved impressive face recognition performance, the research of sparse representation-based face recognition was largely boosted and lots of algorithms have been developed. Gao et al. [7] proposed an extension of SRC named kernel SRC (KSRC), which performed the sparse representation technique in a new high-dimensional feature space obtained by the kernel trick [8]. Meanwhile, Yang et al. [9] utilized Gabor features rather than the original facial features in SRC to improve recognition accuracy. Wang et al. [10] developed a Locality constrained Linear Coding (LLC) scheme. In LLC, the query image is represented only using the nearest codewords (or training samples). However, SRC, KSRC and LLC did not take the structure of the training data into consideration. Thus, their methods may fail to deal with the data which lie on multiple low-dimensional subspaces in the high-dimensional ambient space [11], [12]. In order to overcome this limitation, Elhamifar et al. put forward a structured sparse representation algorithm in [13]. The main idea of their algorithm is to find a good representation of the query sample using the minimum number of structure blocks in the training set. In [14], Wagner et al. proposed a sparse representation-based method that could deal with face misalignment and illumination variation. Yang et al. introduced a Robust Sparse Coding model (RSC) in [15]. RSC relaxed the assumption that the representation residual should follow the Gaussian or Laplacian distribution in original SRC, and sought for a maximum likelihood estimator (MLE) solution for the sparse coding problem. Moreover, Deng et al. [16] proposed another extended SRC (ESRC) method in which they assumed the intra-class variations of one subject can be approximately represented by a sparse linear combination of the other subjects. Therefore, ESRC can successfully handle face recognition with limited training samples per subject. Recently, Mi et al. [17] presented a novel face recognition method named sparse representation-based classification on *k*-nearest subspace (SRC-KNS). In SRC-KNS, the distance between the test image and the subspace of each individual class is first exploited to determine the *k* nearest subspaces, and then the SRC is performed on the *k* selected classes.

Although the above-mentioned SRC-based methods perform well, their recognition performances may also be affected by some problematic factors (such as illumination, expression, disguises and pose) in real-world face images [18]. The main reason is that they utilize the holistic information of the face images for recognition. Based on the observation that some of the local facial features would not vary with pose, lighting, facial expression and disguise, some local matching-based methods which extract facial features from different levels of locality have been proposed showing more promising results in face recognition tasks [3], [19]–[25]. In [6], Wright et al. also incorporated their SRC into the local matching framework to improve its performance. In their local matching-based SRC (LMSRC), the query and training face images are first divided into a number of equally sized sub-images. Then, each sub-image of the query face is represented by the corresponding sub-images of the training set using SRC, and a final decision is made by majority voting for classification. However, since LMSRC represented the sub-images independently, it merely focused on how to sparsely encode each sub-image of the query face but ignored the latent relationships among the multiple sub-images from the same face image, which may weaken its recognition performance [25], [26].

In the local matching-based face recognition framework, each sub-image of a face can be regarded as a sub-pattern which contains a partial feature of the face image. Furthermore, different sub-images divided from the same image can reflect various kinds of information of the query face, and have some latent connections with each other since they jointly provide the full information of the whole face image. Therefore, jointly estimating the sparse representation models of latently-related sub-images from a query face image can be viewed as a “multi-task learning” problem in which each sub-image is a task [27]. Nowadays, some multi-task learning-based sparse representation algorithms have been proposed to take advantage of an object's different features. Yuan et al. proposed a Multi-task Joint Sparse Representation-based Classification method (MTJSRC) in [28]. MTJSRC assumes that the sparse representation coefficients of different features have the same sparsity pattern. Thus, the *ℓ*_1,2_-norm is utilized in this method to make the sparse representation coefficients of different features be the same at atom-level. However, this assumption is too strict to hold in practice. For example, if the appearance of a face image is affected by large illumination changes, it would be hard to represent all sub-images of this face properly by the same set of atoms. Therefore, Zhang et al. proposed a new method named Joint Dynamic Sparse Representation-based Classification (JDSRC) to deal with this problem [29]. In their method, a novel concept of joint dynamic sparsity is introduced to represent the different features of an object by different sets of training samples from the same class [29]. As a result, the sparse representation coefficients of different features obtained by JDSRC tend to have the same sparsity pattern at class-level rather than atom-level. Additionally, another method named Relaxed Collaborative Representation (RCR) was proposed by Yang et al. [27]. RCR assumes the sparse representation coefficients with respect to different features should be alike. Therefore, the sparse representation coefficients of all features obtained by RCR have the similar sparsity pattern in appearance (i.e. the positions and values of non-zero elements in different representation coefficient vectors are similar to each other). Though the experiments in [27]–[29] showed that the MTJSRC, JDSRC and RCR algorithms achieved better recognition and classification performances than SRC, the locality information (i.e. similarity between the query and training samples) [10] was neglected in all of them. Therefore, these algorithms may select the training samples which are dissimilar to the query sub-image for representation and produce unsatisfying recognition results. In recent studies, since the researchers have shown that exploiting locality information of data was more essential than sparsity in some cases [30]–[32], it is crucial to incorporate the locality information into the multi-task learning-based sparse representation algorithms.

Inspired by the pioneer work of joint sparse representation and the importance of data locality, we present a novel multi-task learning-based sparse representation algorithm called Locality Constrained Joint Dynamic Sparse Representation-based Classification (LCJDSRC) for local matching-based face recognition. One important advantage of the proposed algorithm is that it explicitly integrates the joint sparse representation and the locality constraint into a unified framework. Therefore, our algorithm not only takes the latent correlations among different local facial features into account but also considers the similarity between the query and training samples. Like the existing JDSRC, the sparse representation coefficients of different sub-images from a face have the same sparsity pattern at class-level in LCJDSRC. However, since the locality constraint in our algorithm will magnify the representation coefficients corresponding to the similar samples of the query sub-image while reducing the dissimilar ones, LCJDSRC tends to select the nearest neighbors of the query sub-image for representation to improve its recognition performance. The effectiveness of our algorithm is evaluated by extensive experiments on four well-known face databases and compared with other state-of-the-art approaches.

The rest of this paper is organized as follows. The LCJDSRC model is presented in Section 2. In Section 3, the proposed algorithm and several other methods are evaluated on the four databases (ORL, Extended YaleB, AR and LFW). Finally, the conclusions are given in Section 4.

## Locality Constrained Joint Dynamic Sparse Representation

In this section, we first present the outline of the proposed local matching-based face recognition method. Then, the details of our Locality Constrained Joint Dynamic Sparse Representation algorithm are discussed. Next, the recognition criterion of our algorithm is given. Finally, some comparisons between the proposed algorithm and related work are also analyzed.

### Outline

**Figure 1:**
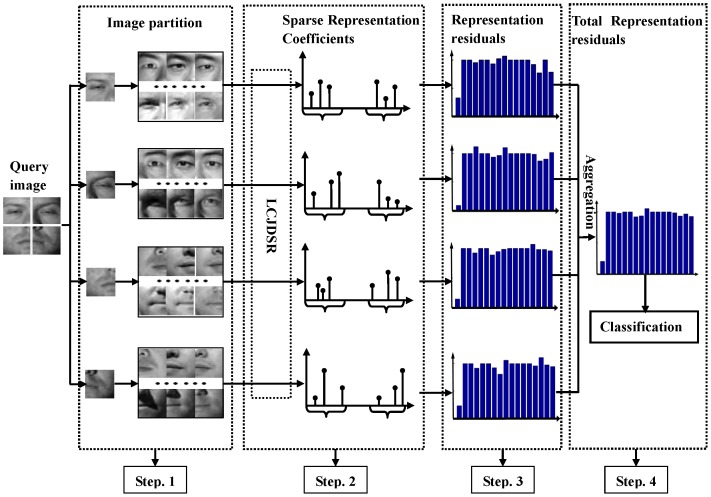
The diagram of the proposed algorithm. The face images are collected from the Extended YaleB database [36].

There are four steps in the proposed local matching-based face recognition algorithm. The first step is to partition the query face image and the face images in the training set. Generally, there are two different techniques to implement the partition, i.e., local components and local regions. Local components are areas occupied by the facial components, such as eyes, nose and mouth, and local regions are local sub-images centered at designated coordinates of a common coordinate system. Since some researchers have verified that the local region partition is to be preferred in local matching-based face recognition [3], we adopt rectangular regions to partition the images as many other approaches do [20]–[25]. That is, the query and training face images are divided into several smaller rectangular sub-images in our algorithm. In the second step, the sub-images of the query face are sparsely represented by their corresponding sub-images in the training set using the proposed locality constrained joint dynamic sparse representation (LCJDSR) algorithm, which not only considers the latent relationships among the sub-images but also takes the locality information into account. The third step of our algorithm is to compute the representation residual of each sub-image using the sparse representation coefficients obtained by LCJDSR. At last, the total representation residuals of all sub-images from the query face are aggregated for final recognition. The flow diagram of the proposed algorithm can be seen in Figure 1.

### Locality Constrained Joint Dynamic Sparse Representation Model

Let 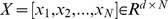
denote *N* face images belonging to *C* persons in the training set (*N_c_* samples, 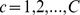 are associated to each person), and let the size of each face image be 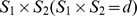. Given a query face image 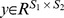, we partition it into *M* non-overlapping sub-images and then concatenate each sub-image into a column vector. Thus, *y* can be represented as 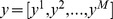 in which 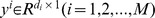 is the vector of the *i-*th sub-image. Similarly, the face images in the training set are also partitioned into 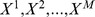, where 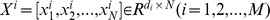 is the set which contains the *i-*th sub-image vectors of all training samples. Here, *d_i_* is the dimension of the *i-*th sub-images in *y* and 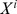.

After partition the query and training face images into non-overlapping sub-images, our objective is to sparsely represent the sub-images in *y* using their corresponding sub-images in the training set. To address this problem, one can simply apply the standard SRC to each of the *M* sub-images in *y*, which can be written as:
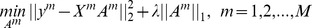
(1) where 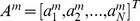 is the coefficient vector of the *m-*th sub-image and 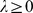 is a tradeoff parameter. Taking all *M* sub-images into account, the objective function of local matching-based SRC is:
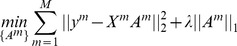
(2)


In fact, this strategy is the same as Wright et al.'s [6]. Therefore, as we have discussed in Section 1, it is far from optimal due to the following reasons. First, the objective function in Equation (2) neglects the similarity between the query and the training samples, thus it may select training sub-images which are not similar to the query face for representation. Second, Equation (2) represents each sub-image of the query face independently, ignoring the latent relationships among the sub-images.

In SRC and other related algorithms, the classification result of a query sample is always determined by the class-wise minimum representation residual [6]–[7], [9]-[10], [13]–[17], so it is reasonable to believe that selecting training samples similar to the query image for representation can improve classification and recognition [30], [31]. According to the above analysis, a locality adaptor which measures the similarity between the query and training samples is introduced in this study to overcome the first shortcoming of Equation (2). The locality adaptor is defined as:
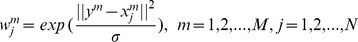
(3) where 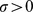 is a parameter which determines the decay rate of the weight function, 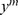 is the *m-*th sub-image of the query face and 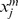 denotes the *m-*th sub-image of the *j-*th training sample. From Equation (3), it is clear that a smaller 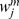 indicates that 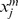 is more similar to the query sub-image 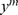, and vice versa.

**Figure 2:**
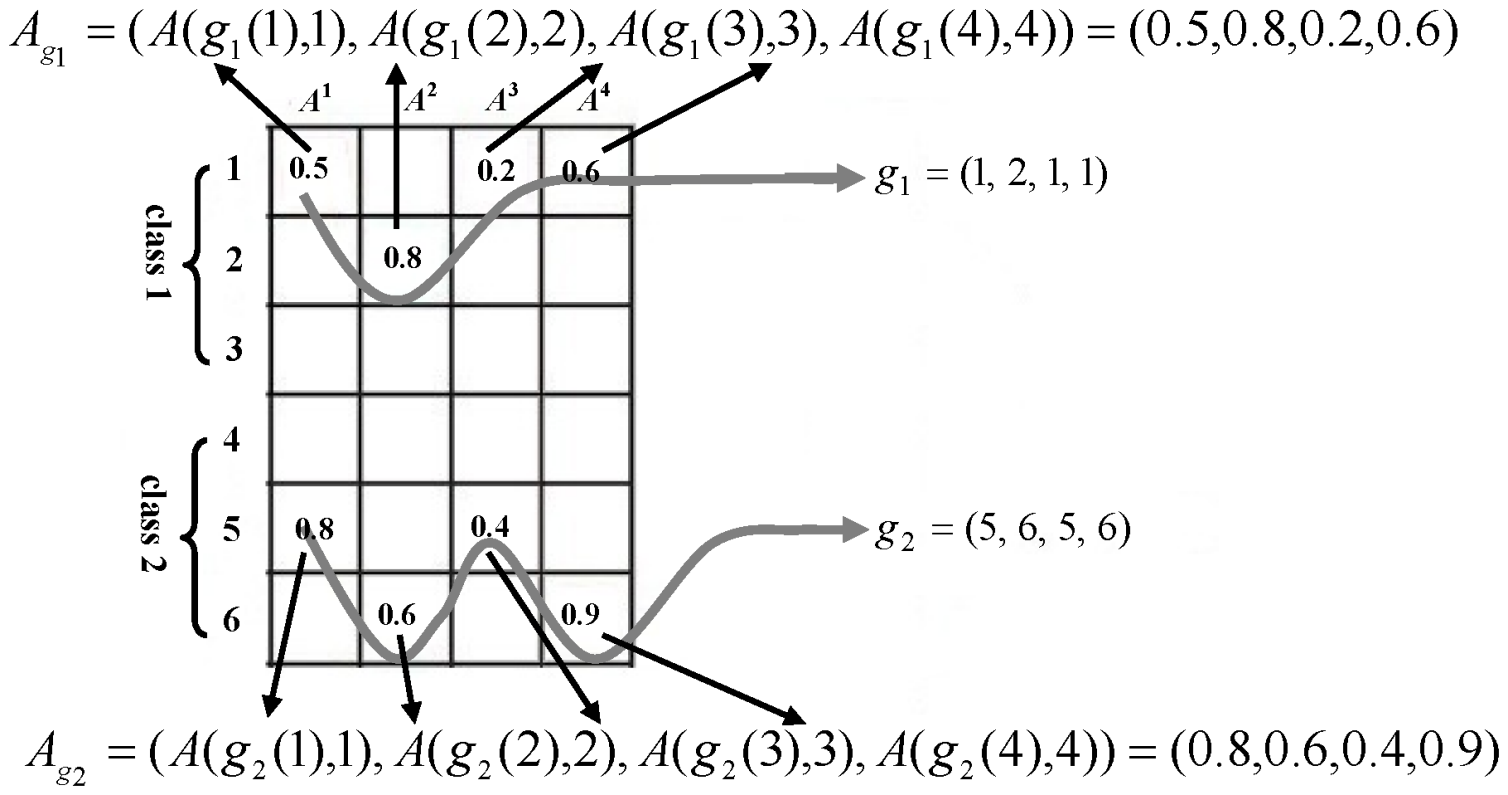
An example of the organization of the dynamic active set. For simplicity, we just take two classes as an example and the number of sub-images *M* is 4. *A* = [*A* ^1^,*A* ^2^,*A* ^3^,*A* ^4^] is a coefficient matrix and *A^i^* (*i* = 1,2,3,4) is a column vector of *A*, the number in each squared block denotes the sparse representation coefficient and the blank blocks denote zero values.

In order to overcome the second problem of Equation (2), a dynamic active set is adopted in our algorithm. The concept of dynamic active set was first proposed by Zhang et al. to exploit the correlations (or relationships) among the multiple observations which describe the same subject during the multi-task learning-based sparse representation. In [29], a dynamic active set is defined as a set of coefficient indices belonging to the same class, and a number of dynamic active sets are jointly activated to sparsely represent the multiple observations. Formally, let 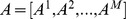 be the matrix containing the sparse representation coefficients of *M* sub-images from *y*, where 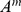 is the coefficient vector of the *m-*th sub-image. Then, each dynamic active set (denoted by 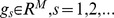) can be described as a set of row indices of coefficients whose corresponding samples in the training set are from the same class. To promote sparsity and allow only a small number of dynamic active sets to be involved during the joint sparsity representation, a mixed-norm which applies *ℓ_2_*-norm on each dynamic active set and then *ℓ*_0_-norm across the *ℓ*_2_-norm is defined as: 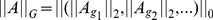
(4) where
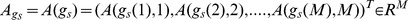
(5) is the vector formed by the coefficients associated with the *s-*th dynamic active set 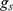, in which 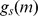 is the row index of the selected training sample for the *m-*th column of *A* in the *s-*th dynamic active set. In order to better illustrate the organization of the dynamic active set, an example is provided in Figure 2. From Figure 2, we can see that there are two dynamic active sets denoted by 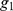 and 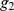 in this example. Therefore, according to the definition of the dynamic active set, we can get 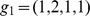 and 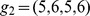. Furthermore, according to Equation (5), the coefficient vectors associated with 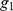 and 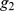 are 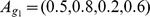 and 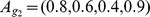, respectively. For more details about the dynamic active set, the readers can refer to [29].

Now, by integrating the locality constraint and the joint sparse representation into a unified framework, we can obtain the objective function of our model as follow
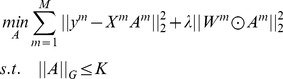
(6) where 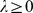 is a parameter to control the tradeoff between the two terms in Equation (6), ⊙denotes the element-wise multiplication, 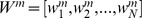 is the locality adaptor vector of the *m-*th sub-image and *K* is the sparsity level which denotes the number of non-zero elements in each 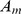 [6], [29].

In the proposed algorithm, since we want to guarantee that the sub-images of a query face can be well represented by their corresponding samples in the training set, the first term in Equation (6) which stands for the representation residual should be minimized. On the other hand, the second term is referred to as locality constraint because minimizing this term will magnify the absolute values of coefficients corresponding to the training samples similar to the query sub-image and reduce the dissimilar ones. Furthermore, the mix-norm regularization term 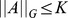 combines the cues from all the sub-images coming from the query image *y* during the representation process and promotes a joint sparsity pattern shared at class-level [29].

### The optimization stage

Since the regularization term in our proposed model contains the *ℓ*_0_-norm, how to solve Equation (6) becomes a challenging problem. In this subsection, a greedy algorithm based on Matching Pursuit (MP) [33] is presented to optimize the objective function in Equation (6). In our algorithm, we initialize the representation residual of each query sub-image as 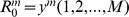 and the selected dynamic active sets *I*_0_ as empty. Then, the following four steps are processed in the *t-*th iteration (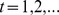) until certain conditions are satisfied.

**Step 1.** Select new candidates based on the current residual.

Based on the current representation residual, some candidate dynamic active sets are selected. First, the representation coefficients of each query sub-image are computed by the inner product of the representation residual and its corresponding training set as:
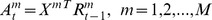
(7)


**Figure 3:**
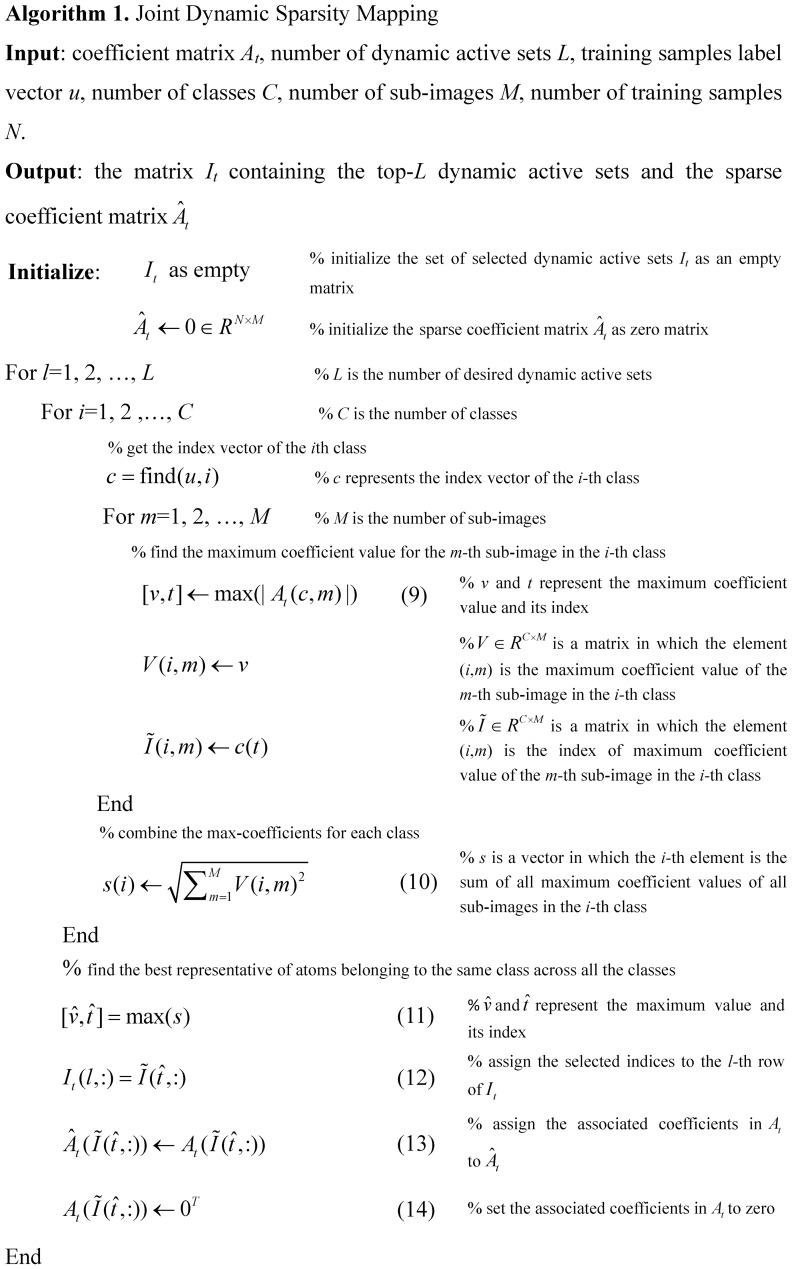
The joint dynamic sparsity mapping algorithm.

**Figure 4:**
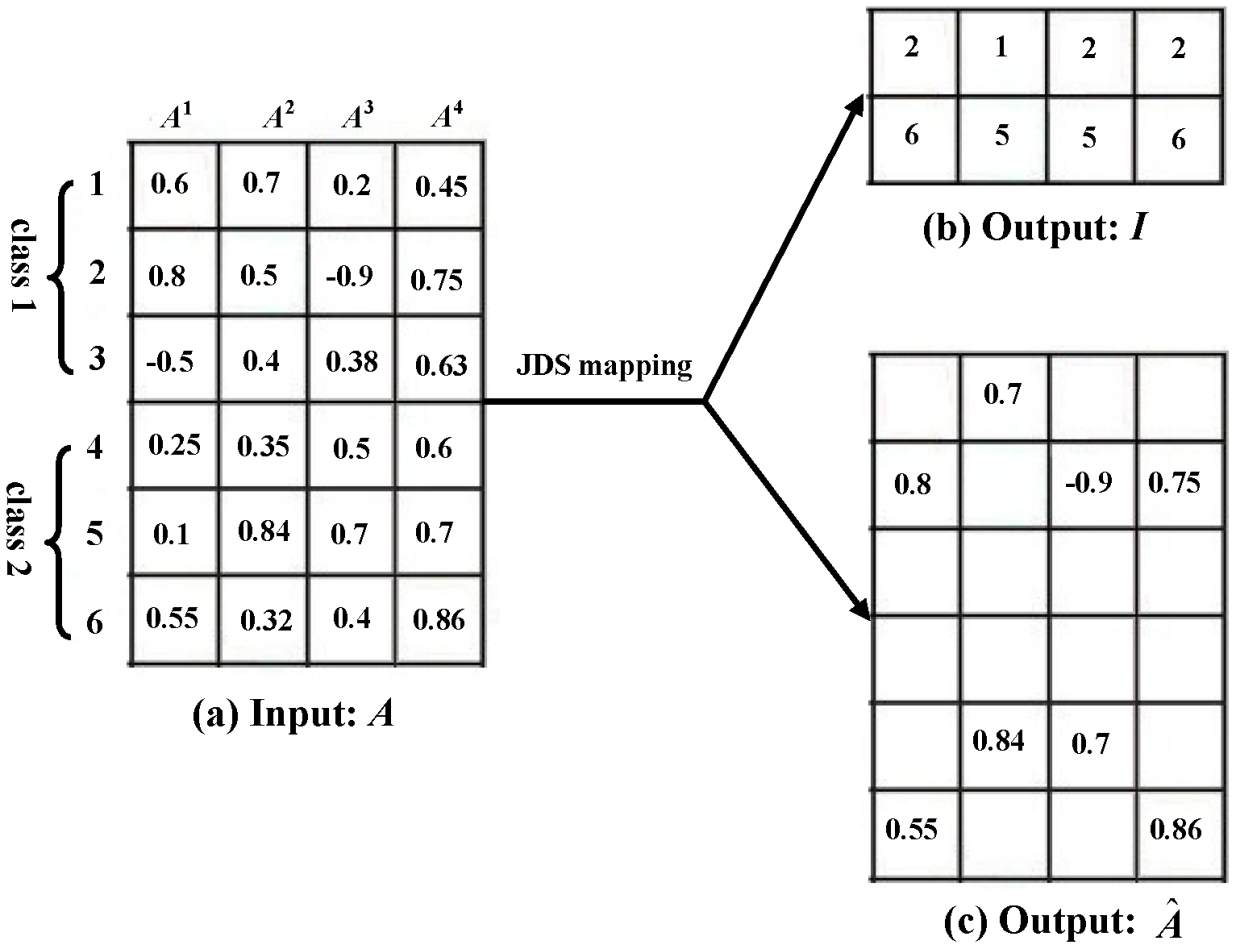
An example about the matrix organization in JDS mapping (*L* = 2). (a) the input representation coefficient matrix *A*; (b) the output matrix *I* contains two selected candidate dynamic active sets selected by JDS mapping; (c) the output sparse matrix 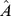 associated with *I*.

Then, according to the representation coefficient matrix 
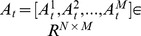
, *L* candidate dynamic active sets whose associated coefficients can best approximate to
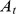
are selected. According to the suggestions in [29] and [34], we set *L* = 2*K* in this study. This problem can be solved by the following objective function:
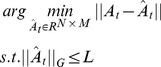
(8) where 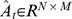 is a sparse matrix which only keeps the coefficients associated with the selected candidate dynamic active sets in 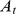 and sets the other coefficients to be zero. The solution of Equation (8) can be obtained by the Joint Dynamic Sparsity mapping (JDS mapping) detailed in Figure 3, which gives the dynamic active sets and associated 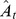 based on the input coefficient matrix [29]. In JDS mapping, one dynamic active set is selected in each of its iteration by four steps. Firstly, the maximum absolute coefficient for each class and each sub-image is calculated by Equation (9). Then, these maximum absolute coefficients are combined across the sub-images for each class as the total response by Equation (10). Third, one of the dynamic active sets which gives the maximum total response is selected by Equation (11). At last, the selected dynamic active sets are added into the matrix *I_t_* as a row and its associated coefficients in 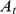 are assigned to 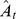 by Equation (12) and (13). In order to ensure that the selected dynamic active set will not be selected again in the following iterations, we also set its associated coefficients in 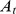 to be zero by Equation (14). These four steps are iterated until the desired number of dynamic active sets is obtained. A simple example about the organizations of matrices *A*, *I* and 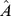 in JDS mapping can be seen in Figure 4.

**Step 2.** Merge the newly selected candidates with the previously selected sets.

After obtaining the matrix *I_t_* which contains the *L* candidate dynamic active sets selected by Algorithm 1, we merge it with *I_t_*_-1_ to update the dynamic active sets:
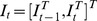
(15)


**Step 3.** Estimate the representation coefficients based on the merged set.

Let 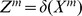 and 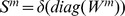, where 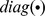 is the vector diagonalization operator and 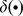 is an operator that only keeps the columns whose indices are included in
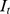
while setting others to be zero vectors in matrices 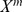 and 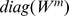. The representation coefficient of each sub-image can be updated by
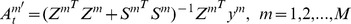
(16)


More details about the derivation process of Equation (16) can be seen in the in the supporting information file S1.

**Step 4.** Prune the merged set to a specified sparsity level based on the newly estimated representation coefficients.

Based on the representation coefficients 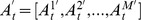 obtained from Equation (16), the *K* most representative dynamic active sets are calculated using JDS mapping. Therefore, the selected dynamic active sets are further updated by
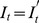
(17) where 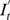 is the matrix which contains the *K* most representative dynamic active sets obtained by JDS mapping. That is, only *K* dynamic active sets in 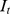 are selected according to 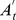 and others are pruned away in this step.

**Step 5.** Update the residual.

Firstly, according to the dynamic active sets 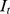 obtained by Step4, the representation coefficient of each sub-image is further obtained by Equation (16). Then, the representation residual of each sub-image is updated by
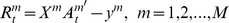
(18)


**Step 6.** Check whether the termination condition is satisfied.

**Figure 5:**
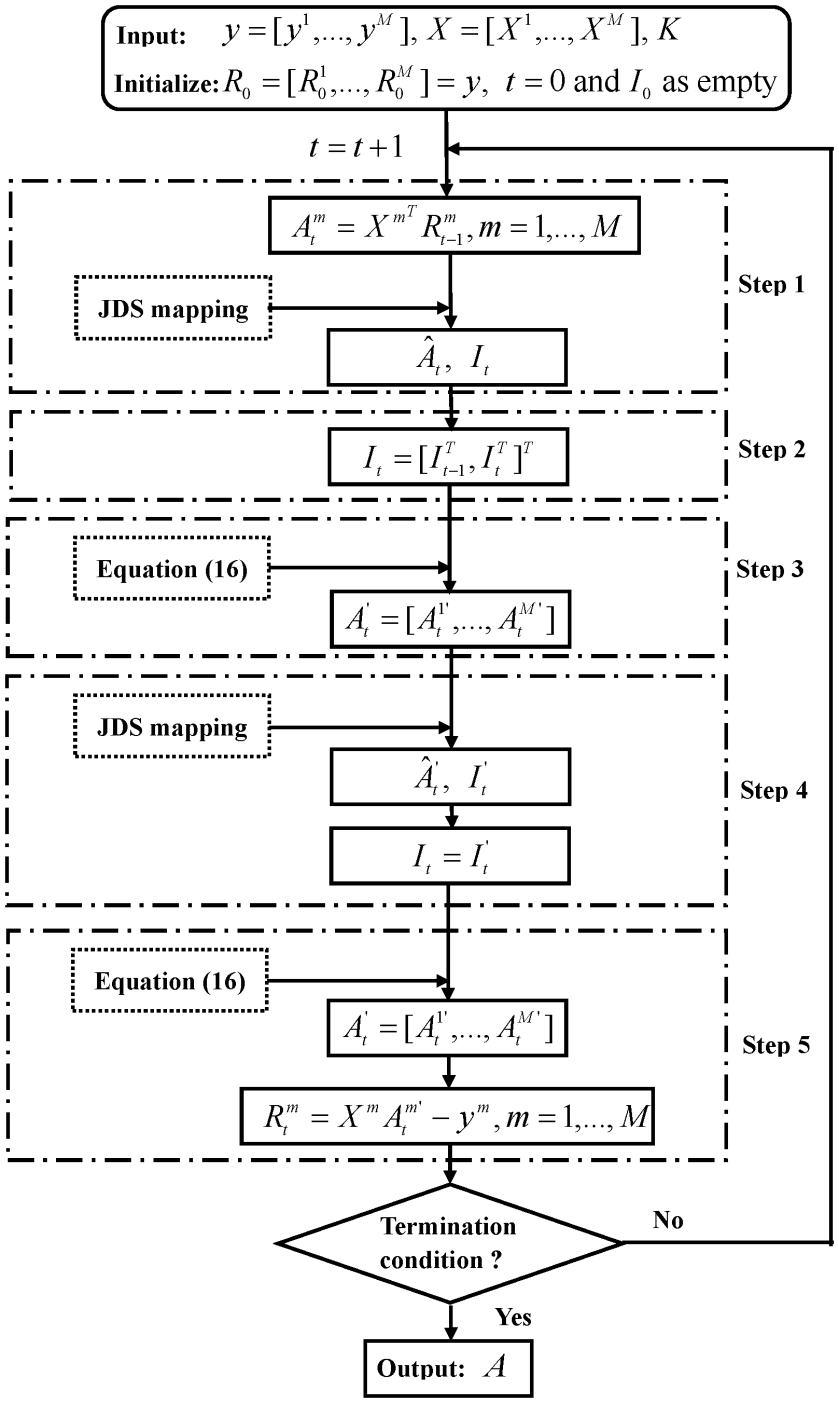
The flowchart of the proposed optimization algorithm.

The termination condition of our algorithm can be defined in two alternative ways. That is, if the predetermined maximum iteration number is reached, or the difference between the representation residuals in adjacent iterations is smaller than a preset value, the algorithm will stop. The flowchart of the proposed optimization algorithm is illustrated in Figure 5.

### Recognition criterion

After obtaining the sparse representation matrix 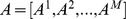, we combine the residuals of all the sub-images in *y* and get the identity of the query face as:
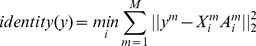
(19) where 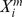 is the subset of 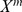 belonging to the *i-*th class, and 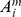 is the coefficient vector of 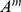 with respect to the *i-*th class 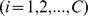.

### Comparisons with other works

In this subsection, the proposed algorithm is compared with other related works to demonstrate its novelty.

**Figure 6:**
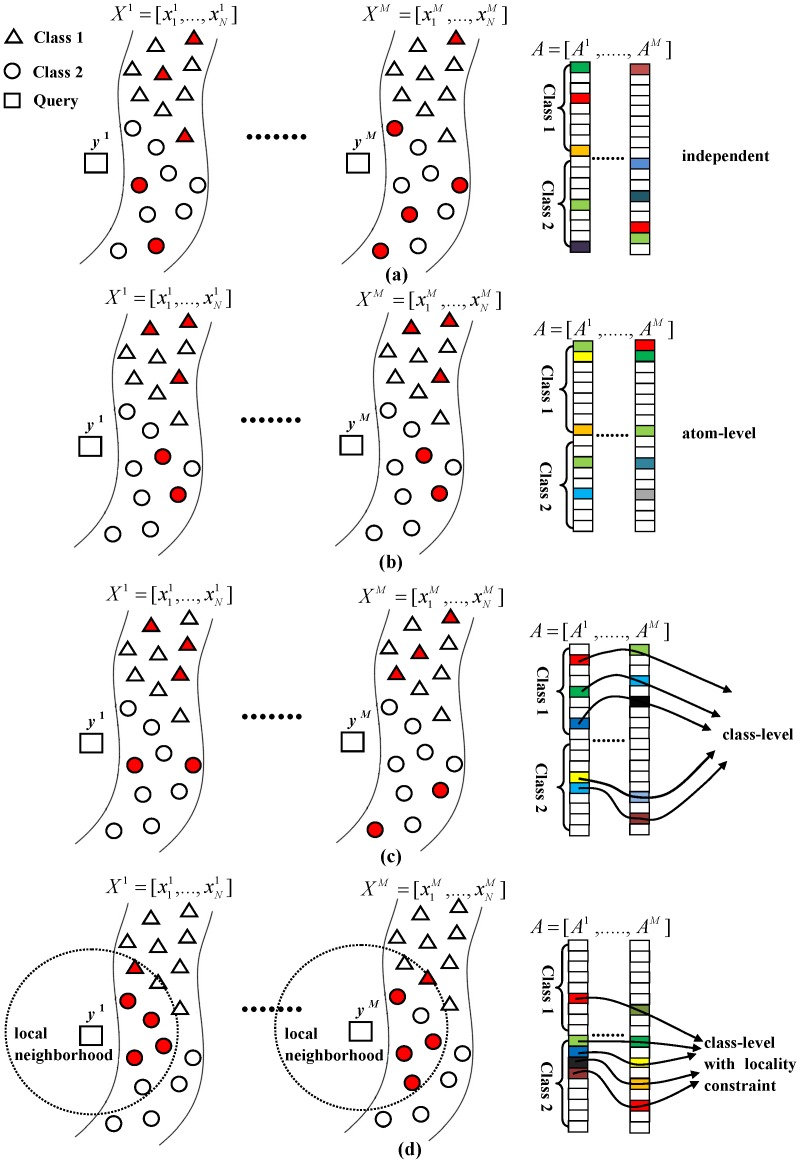
Comparisons among different algorithms. (a) LMSRC, (b) MTJSRC, (c) JDSRC and (d) LCJDSRC. The training sub-images highlighted in red are those selected to represent the query sub-images. In the sparse representation coefficient vectors, the elements marked as "Class 1" and "Class 2" represent the coefficients corresponding to the training samples belonging to each class; the non-zero elements in the vectors are highlighted in colors.

Firstly, the objective function of the proposed algorithm is compared with SRC [6], LMSRC [6], LLC [10], MTJSRC [28] and JDSRC [29]. Since all these methods adopt the sparse representation-based scheme to classify the query samples, it is the regularization on the representation coefficients that makes them different from each other. Specifically, the objective functions of these methods can be written as:
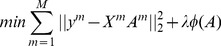
(20) where 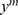 is the *m-*th sub-image of the query face, 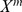 is the *m-*th sub-images of all training samples, 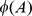 is a regularization term over the representation coefficients 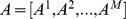 and 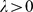 is a tradeoff parameter. When the number of sub-patterns *M* is set to 1, Equation (20) reduces to the holistic classification algorithm. In this case, if we utilize *ℓ*_1_-norm and 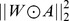 to regularize the representation coefficients, then Equation (20) becomes the standard SRC and LLC. When the number of sub-patterns *M* is larger than 1, if the *ℓ*_1_-norm, *ℓ*_1,2_-norm and
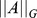
are employed to regularize the representation coefficients, Equation (20) naturally becomes LMSRC [6], MTJSRC [28] and JDSRC [29], respectively. For our algorithm, the regularization 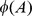 in Equation (20) is 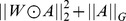. The differences among LMSRC, MTJSRC, JDSRC and LCJDSRC are illustrated in Figure 6. In this figure, the rectangles denote the sub-images of a query face belonging to Class 2, and the triangles and circles represent the training sub-images belonging to Class 1 and Class 2, respectively. From Figure 6a, it can be seen that, since LMSRC simply utilizes the *ℓ*_1_-norm to regularize the representation coefficients, the query sub-images are sparsely represented independently and the representation coefficient vectors (i.e. *A* ^1^,…, *A^M^*) obtained by LMSRC are very different from one another. In MTJSRC, the latent relationships of the sub-images from the query face are considered by *ℓ*_1,2_-norm regularization. Thus, as demonstrated in Figure 6b, if one sub-image of a training face is selected to represent its corresponding sub-image of the query face, then the other sub-images of the same training face will also be selected to represent their corresponding sub-images in *y*. This leads the representation coefficient vectors of different query sub-images obtained by MTJSRC to have the same sparsity pattern at atom-level (i.e. the non-zero elements of different coefficient vectors are located in the same row). For JDSRC and LCJDSRC, since both of them take the latent relationships of the query sub-images into account by employing the mixed-norm of dynamic active set as regularization, the sparse representation coefficient vectors of different query sub-images obtained by these two algorithms have the same sparsity pattern at class-level as shown in Figure 6c and Figure 6d. That is, the non-zero elements in different coefficient vectors joined by each line (i.e. dynamic active set) are from the same class. Furthermore, since the locality information is neglected in LMSRC, MTJSRC and JDSRC, we can find that some distant training samples belonging to Class 1 are selected for representation by these three algorithms in Figure 6a-c, which may result in misrecognition. However, this limitation is overcome by the locality constraint in LCJDSRC. From Figure 6d, it can be seen that by taking the similarity between the query and training sub-images into consideration, the proposed algorithm tends to assign non-zero coefficients to similar training samples within the local neighborhoods of the query sub-images. Therefore, the training sub-images selected for representation by LCJDSRC are mostly from the same class of the query images and the recognition performance can be improved.

Then, the differences between the optimization algorithms in our study and other works are analyzed. Though the optimization algorithm presented in Section 2.3 looks similar to those in CoSOMP [34] and JDSRC [29], there are two key different points between them. First, the dynamic active sets in our algorithm are obtained by Joint Dynamic Sparsity mapping, thus, our algorithm can jointly represent the sub-images of the query face and make the sparsity of the representation coefficients for different sub-images be the same at class level, which is the major difference between our algorithm and CoSOMP. Second, our algorithm updates the representation coefficients using Equation (16). Therefore, the similarity between the query and training samples is considered. However, JDSRC updates the coefficients using standard least squares regression. Thus, LCJDSRC can achieve better recognition result than the JDSRC algorithm.

## Experimental Results and Analysis

In this section, extensive experiments are conducted to verify the effectiveness of the proposed algorithm on four benchmark face databases including ORL [35], Extended YaleB [36], AR [37] and LFW [38]. We compare the performance of our proposed method with four state-of-the-art algorithms, i.e., LMSRC [6], RCR [27], MTJSRC [28] and JDSRC [29]. For all face images in each database, we first normalize them in scale and orientation such that eyes are always in the same position, and then crop the facial areas into the final images for recognition. In order to prevent overfitting and fairly compare our algorithm with other algorithms, we randomly split the samples of each database into three disjoint subsets: a training set used to train different recognition algorithms, a validation set for optimizing the parameters in each algorithm and a test set used to assess the recognition performances of various algorithms.

In local matching-based face recognition methods, a face image can be partitioned into a set of equally or unequally sized sub-images, depending on the user's option. However, how to choose the sub-image size which gives optimal performance is still an open problem. In this work, we will not attempt to deal with this issue. So without loss of generality, equally sized partitions are adopted in our study as in many other approaches [19]–[25].

### Experimental results on the ORL database

In this subsection, we apply the proposed algorithm to the ORL face database which contains 400 face images of 40 individuals, i.e., 10 images per individual. The images were captured under different lighting conditions, facial expressions (open or closed eyes, smiling or not smiling) and facial details (glasses or without glasses). In our experiments, all images are resized to the resolution of 64×64 pixels with 256 gray levels for computation efficiency. In this database, 4 and 3 images of each person are randomly selected for the training and validation sets, and the remaining samples are regarded as the test set. The random sample selection is repeated 10 times.

In a first experiment, the impacts of the two parameters (*K* and 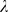) on the performance of our algorithm under different sub-image sizes are evaluated on the validation set. Here, the sub-image size is set as 32×32, 21×32, 16×32 and 21×16. For the parameters *λ* and *K*, we tune their values by searching the grid {0.001, 0.01, 0.05, 0.1, 1, 10, 100, 1000}×{5, 10, 15, 20, 25, 30} (where × is the Cartesian product). From the validation results in Tables S1-S4 in File S1, the optimal parameter values for which our algorithm gives the best performances for various sub-image sizes can be easily found. Moreover, two other interesting points can also be observed. Firstly, when the *λ* value is fixed, a larger *K* will deteriorate the performance of our algorithm. The reason for this phenomenon is that when the sparsity level *K* is large, more training sub-images from the incorrect classes are selected to represent the query sample, thus the recognition rate is reduced. Secondly, we can see that when the sparsity level *K* is fixed, the performance of our algorithm improves as the value of *λ* increases when the *λ* value is relatively small. However, this trend is not maintained for all *K* values. For the cases of *K* = 5, 10, 15 and 20, the performance of our algorithm will slightly decrease after it achieves its top recognition rate. Furthermore, we can find that a larger *λ* value is more suitable for larger sparsity level. This means that when the number of training samples selected for representation becomes large, it is preferable to magnify the coefficients corresponding to training samples similar to the query sub-image and penalize the dissimilar ones, since similar training samples are more likely belong to the same class of the query sample. At last, we can see that given the standard deviation, the differences among the recognition results of LCJDSRC under a large number of parameter sets are not significant. This indicates the proposed algorithm is not sensitive to the parameters when they are set as appropriate values.

In the second experiment, the performance of our algorithm is compared with other algorithms on the test set. According to the validation results in Tables S1-S4 in File S1, the parameters *K* and *λ* in LCJDSRC are set as {*K* = 10, *λ* = 1}, {*K* = 10, *λ* = 1}, {*K* = 10, *λ* = 10} and {*K* = 10, *λ* = 100} for the sub-image size 32×32, 21×32, 16×32 and 21×16, respectively. We also optimized the parameters of the other algorithms in the same manner as for our algorithm. The average recognition results of the algorithms under evaluation over ten independent runs for each experiment can be seen in Table 1. The table shows that LMSRC obtains the worst recognition result among all algorithms. This is because it processes the sub-images of the query face independently. The performances of MTJSRC, JDSRC and RCR are better than LSMRC, since the latent relationships among sub-images are taken into account. For LCJDSRC, we can see that it outperforms all other algorithms, which confirms that both data locality and the joint sparse representation are important to improve face recognition performance. Moreover, the table shows that the recognition performances of all algorithms vary with the sub-image size. For LMSRC, it achieves the best performance under smaller sub-image size. Instead, a larger sub-image size is preferable for MTJSRC, RCR, JDSRC and LCJDSRC. These results show that the proposed algorithm consistently yields better recognition rates than the other algorithms regardless of the sub-image sizes.

**Table 1:** The average recognition rates (%) and the corresponding standard deviations (%) of different algorithms under various sub-image sizes on the test set of the ORL face database.

Size	LMSRC	MTJSRC	RCR	JDSRC	LCJDSRC
32×32	83.25±1.68	85.41±2.05	87.91±2.12	88.42±1.78	91.92±1.80
21×32	82.00±1.97	85.00±2.69	86.58±2.37	86.75±3.21	90.50±2.16
16×32	83.41±2.23	84.66±2.39	86.75±2.30	87.00±2.42	90.67±2.38
16×21	83.08±2.48	84.75±2.54	86.41±1.88	86.66±1.92	89.00±1.66

Finally, in order to further demonstrate the superiority of our algorithm to the other algorithms, the one-tailed Wilcoxon rank sum test is utilized in this study to verify whether LCJDSRC performs significantly better than the other algorithms. In this test, the null hypothesis is that LCJDSRC makes no difference when compared to the other local matching-based algorithms, and the alternative hypothesis is that LCJDSRC makes an improvement when compared to the other algorithms. For example, if we want to compare the performance of our algorithm with that of LMSRC (LCJDSRC vs. LMSRC), the null and alternative hypotheses can be defined as H_0_:  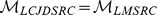 and H_1_: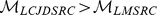, where 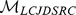 and 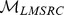 are the medians of the recognition rates obtained by LCJDSRC and LMSRC. In our experiments, the significance level is set to 1%. From the test results in Table 2, we can find that the *p*-values obtained by all pairwise Wilcoxon rank sum tests are much less than 0.01, which means the null hypotheses are rejected in all pairwise tests and the proposed algorithm significantly outperforms the other algorithms.

**Table 2:** The *p*-values of the pairwise one-tailed Wilcoxon rank sum tests on the test set of the ORL database.

	32×32	21×32	16×32	16×21
LCJDSRC vs. LMSRC	8.34e-05	8.73e-05	1.78e-04	1.75e-04
LCJDSRC vs. MTJSRC	9.65e-05	4.80e-04	3.04e-04	3.96e-04
LCJDSRC vs. RCR	3.94e-04	1.03e-03	1.68e-03	2.45e-03
LCJDSRC vs. JDSRC	9.90e-04	1.32e-03	2.18e-03	8.02e-03

### Experimental results on the Extended YaleB database

In this subsection, the performance of the proposed algorithm is evaluated using the Extended YaleB face database, which contains 2414 frontal views of face images from 38 individuals. For each individual, about 64 images were taken under various laboratory-controlled lighting conditions. In our experiment, all images are cropped and resized to the resolution of 64×64 pixels. We randomly select 10 images as training set, 20 images as validation set and the remaining images as test set for each person. This random selection operation is repeated 10 times.

The performances of the proposed algorithm under different parameters values on the validation set are tested firstly. In this experiment, the sub-image size is set as 32×32 and 21×32. Since the number of training samples in this database is much larger than ORL, we tune the values of *K* and *λ* by searching the grid {30, 40, 50, 60, 70, 80, 90}×{0.001, 0.01, 0.05, 0.1, 1, 10, 100, 1000} (where × is the Cartesian product). Tables S5 and S6 in File S1 show the average recognition results of our algorithm under different parameter values. From these tables, it can be found that with the increase of sparsity level, the recognition performance of the proposed algorithm is generally improved. What's more, we can also observe that when the value of sparsity level is small, LCJDSRC performs better under smaller *λ* values, while a relatively larger *λ* is preferred at large sparsity levels. Finally, LCJDSRC achieves its best recognition results as 98.01% and 98.20% when the parameters are set to {*K* = 80, *λ* = 100} and {*K* = 80, *λ* = 10} for the sub-image size 32×32 and 21×32, respectively.

Secondly, we assess the performance of LCJDSRC and compare it with LMSRC, MTJSRC, RCR and JDSRC on the test set. The parameter values for all algorithms are set according to their optimization results on the validation set. In our algorithm, the sparsity level and tradeoff parameter are set as {*K* = 80, *λ* = 100} and {*K* = 80, *λ* = 10}. From the average recognition rates and standard deviations of different algorithms obtained by ten independent repetitions of the experiment reported in Table 3, it can be seen that LCJDSRC outperforms the other algorithms. Furthermore, we can find that the performances of multi-task learning-based algorithms (MTJSRC, RCR, JDSRC and LCJDSRC) are all better than LMSRC. These two observations are consistent with the results obtained on the ORL database. Besides, the one-tailed Wilcoxon rank sum test is also utilized to verify whether the performance of the proposed algorithm is significantly better than the existing algorithms. The *p*-values of the pairwise one-tailed Wilcoxon rank sum tests are listed in Table 4. From these results, we can see that our algorithm significantly outperforms the other algorithms.

**Table 3:** The average recognition rates (%) and the corresponding standard deviations (%) of different algorithms under various sub-image sizes on the test set of the Extended YaleB face database.

Size	LMSRC	MTJSRC	RCR	JDSRC	LCJDSRC
32×32	88.51±1.68	91.13±1.10	92.89±1.40	93.35±1.02	96.67±1.04
21×32	87.30±1.51	90.73±1.20	92.55±1.08	92.41±1.29	95.48±0.97

**Table 4:** The *p*-values of the pairwise one-tailed Wilcoxon rank sum tests on the test set of the Extended YaleB database.

Size	LCJDSRC vs. LMSRC	LCJDSRC vs. MTJSRC	LCJDSRC vs. RCR	LCJDSRC vs. JDSRC
32×32	9.08e-05	9.08e-05	1.64e-04	8.98e-05
21×32	9.13e-05	9.08e-05	1.22e-04	1.23e-04

### Experimental results on the AR database

In this section, we evaluate the performance of our algorithm using the AR database. This database consists of more than 4000 frontal images from 126 subjects including 70 men and 56 women. For each subject, 26 images were taken under different conditions, including illumination, expression, and facial occlusion/disguise. In our experiments, we select a subset which contains 50 males and 50 females from this database. All images are cropped and resized to the resolution of 64×64 pixels.

In the first experiment, 14 images of each individual with only illumination and expression changes are selected. Among these images, 6 images from each person are randomly chosen for training, 4 images are used for validation and the remaining images are utilized for testing. This random selection operation is also repeated 10 times. Similar to Section 3.2, we first set the sub-image size as 32×32 and 21×32, and then find the optimal parameter values of the proposed algorithm using the validation set. From the results in Tables S7 and S8 in File S1, it can be found that the optimal values of *K* and λ for sub-image size of 32×32 and 21×32 are {*K* = 35, *λ* = 0.1} and {*K* = 30, *λ* = 0.1}, respectively. Furthermore, we can also see that the influence of parameter values on the performance of LCJDSRC is consistent with the observations in Section 3.1 and 3.2. Next, the recognition results of our algorithm are compared to the other approaches on the test set. Here, the optimal parameter values in LMSRC, MTJSRC, RCR and JDSRC are obtained in the same way as for LCJDSRC. The average recognition rates obtained by 10 independent runs for each experiment in Table 5 shows that our algorithm outperforms the other algorithms, which is consistent with the experimental results in Section 3.1 and 3.2. However, we can see that our algorithm performs better under the smaller sub-image size, which is opposite to the experimental results on the ORL and Extended YaleB databases. This may happen because the expression variance in this database is much larger than those in the ORL and Extended YaleB, thus a smaller sub-image size is preferable to capture the local facial features which do not vary with the facial expressions. Finally, the *p*-values of pairwise one-tailed Wilcoxon rank sum tests in Table 6 show that the recognition performance of the proposed algorithm is significantly better than the other algorithms.

**Table 5:** The average recognition rates (%) and the corresponding standard deviations (%) of different algorithms under various sub-image sizes on the test set of the AR face database.

	LMSRC	MTJSRC	RCR	JDSRC	LCJDSRC
32×32	90.62±1.35	92.90±1.75	95.07±1.21	94.42±0.63	97.68±0.51
21×32	91.82±1.33	94.05±1.56	95.80±1.08	94.95±0.87	97.90±0.70

**Table 6:** The *p*-values of the pairwise one-tailed Wilcoxon rank sum tests on the test set of the AR face database.

	LCJDSRC vs. LMSRC	LCJDSRC vs. MTJSRC	LCJDSRC vs. RCR	LCJDSRC vs. JDSRC
32×32	8.93e-05	8.78e-05	8.88e-05	8.83e-05
21×32	8.58e-05	9.82e-05	1.54e-04	8.34e-05

The second experiment on the AR database is run to test the effectiveness of our algorithm under severe occlusion conditions. In this experiment, 1400 images with illumination and expression variations from the database are selected for training, 600 images with sunglasses and scarf occlusions are selected for validation and the other 600 images with sunglasses and scarf occlusions are utilized for testing. We optimize the parameter values of different algorithms using the validation set and then compare their recognition performances on the test set. From the validation results in Tables S9-S12 in the File S1, it is easy to find the best parameter values for the proposed algorithm. From the comparison results of various algorithms obtained by ten independent repetitions of the experiment on the test set in Tables 7 and 8, the following points can be observed. Firstly, it can be found that when the face images are occluded by the sunglasses, the recognition performances obtained by all algorithms are relatively low. However, if the faces are occluded by a scarf, the performances of several algorithms improve. This happens because the sunglasses occlude the eyebrows and eyes in the face, which are proved to be the most important components for face recognition [26]. Secondly, we can see that a smaller sub-image size (21×32) is more suitable for the local matching-based algorithms to deal with the face image with occlusions. Finally, it can be obviously seen that our algorithm outperforms the other algorithms. Furthermore, the superiority of our algorithm can also be proved by the pairwise one-tailed Wilcoxon rank sum test results in Tables 9–10.

**Table 7:** The average recognition rates (%) and the corresponding standard deviations (%) of different algorithms on the test set of the AR face database with sunglasses and scarf occlusions (sub-image size 32×32).

	LMSRC	MTJSRC	RCR	JDSRC	LCJDSRC
Sunglasses	64.30±1.65	70.86±1.87	73.06±1.67	79.13±1.54	81.30±1.13
Scarf	83.13±0.89	85.50±1.05	88.73±1.09	87.76±0.78	90.86±0.84

**Table 8:** The average recognition rates (%) and the corresponding standard deviations (%) of different algorithms on the test set of the AR face database with sunglasses and scarf occlusions (sub-image size 21×32).

	LMSRC	MTJSRC	RCR	JDSRC	LCJDSRC
Sunglasses	83.30±0.89	86.00±1.26	89.20±0.86	90.20±0.75	92.76±0.72
Scarf	87.73±1.06	88.83±1.34	91.40±1.26	91.46±1.11	94.83±0.61

**Table 9:** The *p*-values of the pairwise one-tailed Wilcoxon rank sum tests on the test set of the AR face database with sunglasses and scarf occlusions (sub-image size 32×32).

	LCJDSRC vs. LMSRC	LCJDSRC vs. MTJSRC	LCJDSRC vs. RCR	LCJDSRC vs. JDSRC
Sunglasses	8.83e-05	8.83e-05	8.93e-05	3.58 e-03
Scarf	8.34e-05	8.58e-05	6.32e-05	8.58e-05

**Table 10:** The *p*-values of the pairwise one-tailed Wilcoxon rank sum tests on the test set of the AR face database with sunglasses and scarf occlusions (sub-image size 21×32).

	LCJDSRC vs. LMSRC	LCJDSRC vs. MTJSRC	LCJDSRC vs. RCR	LCJDSRC vs. JDSRC
Sunglasses	7.78e-05	7.92e-05	7.92e-05	7.92e-05
Scarf	8.25e-05	8.20e-05	9.65e-05	8.39e-05

### Experimental results on the LFW database

The LFW database [38] is a large scale database which contains 13,233 target face images of 5,749 different individuals. Since all the samples were taken from the real world in an unconstrained environment, facial expressions, pose, illumination, occlusions and alignment are very variable in this database. As suggested in [39], a subset which contains 1580 face images of 158 individuals from the LFW-a [40] database is employed in our study. In this subset, each individual has 10 images with the size of 32×32 pixel (The mat file can be download from http://www4.comp.polyu.edu.hk/~cslzhang/code/MPCRC_eccv12_code.zip). In our experiment, 6 samples of each individual are randomly selected for training. Among the remaining 4 samples, 2 images of each individual are randomly chosen for validation and the other 2 images are used for testing. This random selection operation is also repeated 10 times. Here, we equally partition the face images into four sub-images and the sub-image size is 16×16.

Firstly, the performances of the proposed algorithm under various parameter values are tested on the validation set to find the optimal parameters for our algorithm. From the validation results in Table S13 in the File S1, we can see that the influence of the two parameters on the proposed algorithm is similar to those in Section 3.1-3.3, and the optimal parameter values for which our algorithm gives the best recognition rate are *K* = 50 and *λ*  = 0.05.

Then, we compare the proposed algorithm with other algorithms on the test set. From the average recognition rate of each algorithm over ten independent runs for each experiment in Table 11 and the Wilcoxon rank sum test results in Table 12, it can be said that, although the recognition performances of all algorithms on the LFW database are relatively lower than those on the other three databases, our algorithm is still significantly superior to the other algorithms.

**Table 11:** The average recognition rates (%) and the corresponding standard deviations (%) of different algorithms on the test set of the LFW face database (sub-image size 32×32).

	LMSRC	MTJSRC	RCR	JDSRC	LCJDSRC
32×32	35.88±1.36	42.97±2.48	44.71±2.23	45.53±2.20	49.34±0.99

**Table 12:** The *p*-values of the pairwise one-tailed Wilcoxon rank sum tests on the test set of the LFW face database (sub-image size 32×32).

	LCJDSRC vs. LMSRC	LCJDSRC vs. MTJSRC	LCJDSRC vs. RCR	LCJDSRC vs. JDSRC
32×32	8.93e-05	8.98e-05	8.93e-05	1.04e-04

## Conclusion and Future Work

In this paper, a novel classification algorithm named Locality Constrained Joint Dynamic Sparse Representation-based Classification (LCJDSRC) has been proposed for local matching-based face recognition. Our algorithm combines the joint sparse representation and locality constraint into a unified framework. Therefore, not only does it consider the latent relationships among different sub-images of a face, but also introduces the locality information into the sparse representation model. Moreover, a greedy algorithm based on Matching Pursuit (MP) has been presented to optimize the objective function of LCJDSRC. Extensive experiments have been carried out on four databases including ORL, Extended YaleB, AR and LFW to demonstrate the effectiveness of our proposed LCJDSRC approach. The experimental results have shown that LCJDSRC outperforms several similar methods such as LMSRC, MTJSRC, RCR and JDSRC on the data sets considered in our tests.

Finally, it should be pointed out that in LCJDSRC, the query sub-images are represented by the sub-images partitioned from the original training samples, which may decrease its performance when too few training samples are available. Thus, one of our future goals is to incorporate our algorithm into the dictionary learning framework [41], [42] to further improve its flexibility. Besides, since some researchers have shown that the dimensionality reduction methods are helpful to the sparse representation-based classification algorithms, how to combine LCJDSRC with the dimensionality reduction techniques is another interesting topic for future study.

## Supporting Information

File S1 **Tables S1-S13 and Text S1.** Table S1. The average recognition rates (%) and the corresponding standard deviations (%) of LCJDSRC under different parameters on the validation set of the ORL face database (sub-image size is 32×32). Table S2. The average recognition rates (%) and the corresponding standard deviations (%) of LCJDSRC under different parameters on the validation set of the ORL face database (sub-image size is 21×32). Table S3. The average recognition rates (%) and the corresponding standard deviations (%) of LCJDSRC under different parameters on the validation set of the ORL face database (sub-image size is 16×32). Table S4. The average recognition rates (%) and the corresponding standard deviations (%) of LCJDSRC under different parameters on the validation set of the ORL face database (sub-image size is 16×21). Table S5. The average recognition rates (%) and the corresponding standard deviations (%) of LCJDSRC under different parameters on the validation set of the Extended YaleB face database (sub-image size is 32×32). Table S6. The average recognition rates (%) and the corresponding standard deviations (%) of LCJDSRC under different parameters on the validation set of the Extended YaleB face database (sub-image size is 21×32). Table S7. The average recognition rates (%) and the corresponding standard deviations (%) of LCJDSRC under different parameters on the validation set of the AR face database (sub-image size is 32×32). Table S8. The average recognition rates (%) and the corresponding standard deviations (%) of LCJDSRC under different parameters on the validation set of the AR face database (sub-image size is 21×32). Table S9. The average recognition rates (%) and the corresponding standard deviations (%) of LCJDSRC under different parameters on the validation set of the AR face database with sunglasses occlusion (sub-image size is 32×32). Table S10. The average recognition rates (%) and the corresponding standard deviations (%) of LCJDSRC under different parameters on the validation set of the AR face database with scarf occlusion (sub-image size is 32×32). Table S11. The average recognition rates (%) and the corresponding standard deviations (%) of LCJDSRC under different parameters on the validation set of the AR face database with sunglasses occlusion (sub-image size is 21×32). Table S12. The average recognition rates (%) and the corresponding standard deviations (%) of LCJDSRC under different parameters on the validation set of the AR face database with scarf occlusion (sub-image size is 21×32). Table S13. The average recognition rates (%) and the corresponding standard deviations (%) of LCJDSRC under different parameters on the validation set of the LFW face database (sub-image size is 32×32). Text S1. The derivation process of Equation (16).
(DOC)
