# Supplementary material for: Locality Constrained Joint Dynamic Sparse Representation for Local Matching Based Face Recognition
Source: PLoS One. 2014 Nov 24;9(11):e113198. doi: 10.1371/journal.pone.0113198 (PMC4242617; doi:10.1371/journal.pone.0113198)
Supplement: File S1 — Tables S1-S13 and Text S1. Table S1. The average recognition rates (%) and the corresponding standard deviations (%) of LCJDSRC under different parameters on the validation set of the ORL face database (sub-image size is 32×32). Table S2. The average recognition rates (%) and the corresponding standard deviations (%) of LCJDSRC under different parameters on the validation set of the ORL face database (sub-image size is 21×32). Table S3. The average recognition rates (%) and the corresponding standard deviations (%) of LCJDSRC under different parameters on the validation set of the ORL face database (sub-image size is 16×32). Table S4. The average recognition rates (%) and the corresponding standard deviations (%) of LCJDSRC under different parameters on the validation set of the ORL face database (sub-image size is 16×21). Table S5. The average recognition rates (%) and the corresponding standard deviations (%) of LCJDSRC under different parameters on the validation set of the Extended YaleB face database (sub-image size is 32×32). Table S6. The average recognition rates (%) and the corresponding standard deviations (%) of LCJDSRC under different parameters on the validation set of the Extended YaleB face database (sub-image size is 21×32). Table S7. The average recognition rates (%) and the corresponding standard deviations (%) of LCJDSRC under different parameters on the validation set of the AR face database (sub-image size is 32×32). Table S8. The average recognition rates (%) and the corresponding standard deviations (%) of LCJDSRC under different parameters on the validation set of the AR face database (sub-image size is 21×32). Table S9. The average recognition rates (%) and the corresponding standard deviations (%) of LCJDSRC under different parameters on the validation set of the AR face database with sunglasses occlusion (sub-image size is 32×32). Table S10. The average recognition rates (%) and the corresponding standard deviations (%) of LCJDSRC under di [file pone.0113198.s001.doc]

**Table S1 The average recognition rates (%) and the corresponding** **standard deviations (%) of LCJDSRC under different parameters on the validation set of the ORL face database (sub-image size is 32×32).**

|  | *K*=5 | *K*=10 | *K*=15 | *K*=20 | *K*=25 | *K*=30 |
| --- | --- | --- | --- | --- | --- | --- |
| *λ=*0.001 | 92.33±1.70 | 91.50±1.02 | 91.83±2.07 | 90.41±1.81 | 89.75±1.80 | 89.58±2.72 |
| *λ=*0.01 | 92.91±1.85 | 92.00±2.08 | 91.41±1.71 | 91.50±1.56 | 91.41±1.41 | 90.25±2.77 |
| *λ=*0.05 | 93.32±1.66 | 93.25±1.49 | 92.41±1.68 | 92.08±1.19 | 91.41±1.57 | 91.83±1.40 |
| *λ=*0.1 | 93.83±1.42 | 93.75±1.53 | 92.50±1.03 | 92.33±1.29 | 92.25±1.57 | 91.83±2.24 |
| *λ=*1 | 93.75±1.48 | 94.75±1.96 | 93.83±1.67 | 93.58±1.41 | 92.58±2.16 | 91.66±1.36 |
| *λ=*10 | 93.50±1.99 | 94.16±1.88 | 94.50±1.97 | 93.83±1.53 | 93.16±2.24 | 93.16±2.44 |
| *λ=*100 | 93.00±1.72 | 94.08±1.59 | 94.08±1.98 | 94.00±1.74 | 93.41±2.52 | 93.50±1.79 |
| *λ=*1000 | 92.91±1.72 | 93.58±1.66 | 94.08±1.77 | 94.25±2.09 | 94.16±2.04 | 93.83±2.49 |

**Table S2** The average recognition rates (%) and the corresponding standard deviations (%) of LCJDSRC under different parameters on the validation set of the ORL face database (sub-image size is 21×32).

|  | *K*=5 | *K*=10 | *K*=15 | *K*=20 | *K*=25 | *K*=30 |
| --- | --- | --- | --- | --- | --- | --- |
| *λ=*0.001 | 90.50±2.72 | 90.16±2.21 | 89.25±1.81 | 87.25±2.60 | 86.08±1.47 | 85.83±3.76 |
| *λ=*0.01 | 90.50±2.58 | 90.33±1.58 | 90.50±2.22 | 90.16±2.18 | 88.7±52.58 | 87.91±2.64 |
| *λ=*0.05 | 92.16±1.42 | 91.83±1.40 | 91.58±1.94 | 91.6±62.32 | 90.08±1.14 | 89.75±2.54 |
| *λ=*0.1 | 92.58±2.16 | 91.50±2.38 | 91.83±1.99 | 92.00±1.85 | 90.68±1.61 | 90.50±2.15 |
| *λ=*1 | 92.41±1.94 | 93.33±2.45 | 92.16±2.26 | 92.00±1.89 | 91.00±2.38 | 90.16±2.35 |
| *λ=*10 | 92.16±2.58 | 93.25±1.98 | 92.25±2.22 | 92.00±2.55 | 91.50±1.99 | 90.50±2.55 |
| *λ=*100 | 91.83±2.10 | 92.58±1.81 | 92.41±2.30 | 91.75±2.16 | 92.08±2.12 | 91.41±2.39 |
| *λ=*1000 | 91.91±2.57 | 92.50±2.11 | 92.83±2.52 | 90.91±2.40 | 92.16±2.39 | 92.00±2.29 |

**Table S3 The average recognition rates (%) and the corresponding standard deviations (%) of LCJDSRC under different parameters on the validation set of the ORL face database (sub-image size is 16×32).**

|  | *K*=5 | *K*=10 | *K*=15 | *K*=20 | *K*=25 | *K*=30 |
| --- | --- | --- | --- | --- | --- | --- |
| *λ=*0.001 | 91.91±1.47 | 90.75±2.55 | 88.83±2.97 | 87.41±2.46 | 86.16±3.29 | 84.00±2.50 |
| *λ=*0.01 | 92.16±2.22 | 90.83±2.32 | 90.41±2.33 | 88.83±3.33 | 87.41±2.33 | 87.08±2.46 |
| *λ=*0.05 | 92.50±2.22 | 91.41±1.66 | 90.91±2.13 | 89.83±2.59 | 88.83±2.12 | 88.50±2.59 |
| *λ=*0.1 | 93.25±1.81 | 91.41±2.51 | 91.41±1.71 | 89.66±1.85 | 89.08±2.02 | 88.33±2.57 |
| *λ=*1 | 92.91±1.72 | 92.58±1.86 | 91.58±2.02 | 91.50±2.65 | 90.08±2.30 | 89.58±1.89 |
| *λ=*10 | 92.91±2.36 | 93.16±1.45 | 92.33±1.70 | 91.33±2.58 | 91.16±2.75 | 89.75±2.48 |
| *λ=*100 | 92.66±1.99 | 92.91±1.12 | 92.58±1.32 | 92.00±1.76 | 91.33±2.81 | 90.41±2.01 |
| *λ=*1000 | 92.66±2.10 | 92.75±1.41 | 92.41±1.59 | 91.83±1.45 | 91.33±2.33 | 90.33±3.40 |

**Table S4 The average recognition rates (%) and the corresponding standard deviations (%) of LCJDSRC under different parameters on the validation set of the ORL face database (sub-image size is 16×21).**

|  | *K*=5 | *K*=10 | *K*=15 | *K*=20 | *K*=25 | *K*=30 |
| --- | --- | --- | --- | --- | --- | --- |
| *λ=*0.001 | 90.33±2.29 | 89.58±1.93 | 85.50±3.14 | 83.66±3.33 | 81.83±3.55 | 81.00±3.80 |
| *λ=*0.01 | 92.25±2.18 | 90.08±2.40 | 88.4±12.05 | 87.33±3.25 | 86.00±2.10 | 85.00±3.09 |
| *λ=*0.05 | 93.00±2.29 | 91.50±1.95 | 89.91±1.38 | 89.66±2.42 | 88.16±3.70 | 86.33±3.40 |
| *λ=*0.1 | 92.58±2.37 | 92.25±2.22 | 91.33±2.55 | 80.25±2.52 | 88.58±2.18 | 86.33±2.61 |
| *λ=*1 | 92.75±1.75 | 92.66±2.68 | 91.91±1.84 | 90.25±2.32 | 88.75±2.12 | 87.33±2.93 |
| *λ=*10 | 92.33±2.35 | 93.16±1.91 | 91.83±2.38 | 90.33±2.67 | 89.33±3.13 | 88.16±3.01 |
| *λ=*100 | 92.83±2.64 | 93.25±2.49 | 92.08±2.26 | 91.50±2.53 | 89.83±2.88 | 88.75±3.38 |
| *λ=*1000 | 93.16±2.38 | 92.83±2.19 | 92.16±3.07 | 91.41±2.35 | 90.08±2.95 | 88.91±2.54 |

**Table S5 The average recognition rates (%) and the corresponding standard deviations (%) of LCJDSRC under different parameters on the validation set of the Extended YaleB face database (sub-image size is 32×32).**

|  | *K*=30 | *K*=40 | *K*=50 | *K*=60 | *K*=70 | *K*=80 | *K*=90 |
| --- | --- | --- | --- | --- | --- | --- | --- |
| *λ=*0.001 | 96.13±0.75 | 96.44±0.67 | 96.64±0.65 | 96.94±0.73 | 96.98±0.60 | 97.29±0.60 | 97.02±0.53 |
| *λ=*0.01 | 96.94±0.60 | 97.36±0.59 | 97.34±0.66 | 97.59±0.61 | 97.55±0.66 | 97.77±0.44 | 97.57±0.54 |
| *λ=*0.05 | 97.43±0.43 | 97.35±0.68 | 97.51±0.53 | 97.75±0.51 | 97.76±0.27 | 97.80±0.41 | 97.61±0.48 |
| *λ=*0.1 | 97.42±0.54 | 97.64±0.53 | 97.65±0.68 | 97.53±0.42 | 97.73±0.55 | 97.76±0.50 | 97.65±0.58 |
| *λ=*1 | 96.72±0.60 | 97.32±0.39 | 97.51±0.28 | 97.71±0.41 | 97.85±0.45 | 97.90±0.36 | 97.77±0.52 |
| *λ=*10 | 95.53±0.59 | 96.47±0.58 | 97.11±0.44 | 97.48±0.57 | 97.81±0.39 | 98.00±0.49 | 97.85±0.41 |
| *λ=*100 | 95.34±0.43 | 96.47±0.61 | 97.02±0.54 | 97.71±0.41 | 97.43±0.31 | 98.01±0.53 | 97.59±0.38 |
| *λ=*1000 | 94.81±0.79 | 96.22±0.71 | 96.73±0.50 | 97.14±0.67 | 97.30±0.53 | 97.56±0.43 | 97.36±0.49 |

**Table S6 The average recognition rates (%) and the corresponding standard deviations (%) of LCJDSRC under different parameters on the validation set of the Extended YaleB face database (sub-image size is 21×32).**

|  | *K*=30 | *K*=40 | *K*=50 | *K*=60 | *K*=70 | *K*=80 | *K*=90 |
| --- | --- | --- | --- | --- | --- | --- | --- |
| *λ=*0.001 | 95.64±0.69 | 95.94±0.70 | 96.32±0.74 | 96.21±0.62 | 96.52±0.69 | 96.97±0.75 | 96.73±0.67 |
| *λ=*0.01 | 96.92±0.61 | 97.35±0.68 | 97.42±0.73 | 97.43±0.47 | 97.44±0.34 | 97.50±0.50 | 97.30±0.63 |
| *λ=*0.05 | 97.35±0.25 | 97.55±0.37 | 97.59±0.43 | 97.61±0.21 | 97.52±0.57 | 97.34±0.57 | 97.29±0.56 |
| *λ=*0.1 | 97.46±0.37 | 97.71±0.34 | 97.51±0.34 | 97.48±0.48 | 97.35±0.51 | 97.43±0.61 | 97.39±0.56 |
| *λ=*1 | 96.98±0.38 | 97.55±0.61 | 97.80±0.38 | 97.77±0.58 | 97.72±0.34 | 97.61±0.47 | 97.55±0.68 |
| *λ=*10 | 95.98±0.93 | 96.85±0.53 | 97.57±0.55 | 97.80±0.46 | 98.01±0.36 | 98.20±0.35 | 97.96±0.34 |
| *λ=*100 | 95.76±0.78 | 96.75±0.59 | 97.38±0.66 | 97.73±0.38 | 97.86±0.34 | 97.99±0.52 | 97.98±0.54 |
| *λ=*1000 | 95.35±0.79 | 96.22±0.71 | 96.81±0.52 | 97.34±0.64 | 97.48±0.62 | 97.50±0.61 | 97.41±0.63 |

**Table S7 The average recognition rates (%) and the corresponding standard deviations (%) of LCJDSRC under different parameters on the validation set of the AR face database (sub-image size is 32×32).**

|  | *K*=10 | *K*=15 | *K*=20 | *K*=25 | *K*=30 | *K*=35 | *K*=40 |
| --- | --- | --- | --- | --- | --- | --- | --- |
| *λ=*0.001 | 93.85±1.60 | 94.47±1.18 | 94.77±0.70 | 94.80±0.87 | 94.95±0.92 | 94.77±1.13 | 95.10±0.84 |
| *λ=*0.01 | 94.75±1.09 | 95.72±0.99 | 95.90±1.28 | 96.25±1.13 | 96.15±1.13 | 96.27±1.08 | 96.12±0.81 |
| *λ=*0.05 | 95.27±0.98 | 95.75±1.33 | 96.12±1.01 | 96.72±1.08 | 96.52±1.07 | 96.32±0.97 | 96.32±1.21 |
| *λ=*0.1 | 95.12±0.95 | 95.57±0.94 | 96.10±1.10 | 96.27±0.99 | 96.37±0.92 | 96.75±1.13 | 96.55±1.09 |
| *λ=*1 | 94.62±1.14 | 95.15±0.82 | 95.92±1.13 | 96.12±1.23 | 96.07±1.29 | 95.97±1.15 | 96.15±1.36 |
| *λ=*10 | 94.55±1.15 | 95.12±1.39 | 95.55±1.35 | 96.00±1.25 | 96.32±1.33 | 96.35±1.03 | 96.55±0.94 |
| *λ=*100 | 94.12±1.18 | 94.72±1.20 | 95.47±1.27 | 95.87±1.39 | 96.15±1.49 | 96.17±1.31 | 96.30±1.12 |
| *λ=*1000 | 93.10±1.10 | 94.00±0.92 | 94.62±1.10 | 95.20±1.91 | 95.35±0.79 | 95.95±1.21 | 96.00±1.09 |

**Table S8 The average recognition rates (%) and the corresponding standard deviations (%) of LCJDSRC under different parameters on the validation set of the AR face database (sub-image size is 21×32).**

|  | *K*=10 | *K*=15 | *K*=20 | *K*=25 | *K*=30 | *K*=35 | *K*=40 |
| --- | --- | --- | --- | --- | --- | --- | --- |
| *λ=*0.001 | 94.77±0.69 | 95.50±0.74 | 96.07±0.57 | 95.95±0.68 | 95.72±0.82 | 95.57±0.80 | 95.80±0.66 |
| *λ=*0.01 | 95.15±1.02 | 95.97±1.13 | 96.27±0.87 | 96.80±0.94 | 96.77±0.62 | 96.62±0.71 | 96.65±0.70 |
| *λ=*0.05 | 95.25±1.14 | 95.90±1.14 | 96.57±0.89 | 96.92±0.90 | 96.70±0.95 | 96.97±0.80 | 96.77±0.80 |
| *λ=*0.1 | 95.52±0.90 | 96.15±0.83 | 96.60±0.93 | 96.62±1.06 | 97.00±0.98 | 96.55±0.75 | 96.90±0.80 |
| *λ=*1 | 94.87±0.94 | 95.70±1.09 | 96.22±0.80 | 96.70±0.70 | 96.75±0.88 | 96.57±0.73 | 96.75±0.86 |
| *λ=*10 | 94.57±1.09 | 95.55±0.82 | 96.02±1.04 | 96.15±1.00 | 96.55±0.84 | 96.55±1.07 | 96.82±0.83 |
| *λ=*100 | 94.30±0.80 | 95.12±0.92 | 96.02±0.86 | 96.30±0.93 | 96.52±0.81 | 96.67±0.95 | 96.60±0.96 |
| *λ=*1000 | 93.50±0.84 | 94.55±1.20 | 95.17±1.00 | 95.62±1.00 | 95.62±1.10 | 96.27±0.84 | 96.30±1.05 |

**Table S9 The average recognition rates (%) and the corresponding standard deviations (%) of LCJDSRC under different parameters on the validation set of the AR face database with sunglasses occlusion (sub-image size is 32×32).**

|  | *K*=10 | *K*=20 | *K*=30 | *K*=40 | *K*=50 | *K*=60 |
| --- | --- | --- | --- | --- | --- | --- |
| *λ*=0.0001 | 76.26±1.87 | 76.93±1.62 | 75.73±1.07 | 79.86±1.13 | 79.13±1.54 | 77.30±1.46 |
| *λ*=0.001 | 77.60±1.29 | 78.86±2.00 | 78.43±1.22 | 82.06±1.00 | 81.30±1.20 | 79.76±1.40 |
| *λ*=0.01 | 77.40±1.61 | 77.56±1.95 | 79.13±1.48 | 78.70±1.36 | 78.40±1.29 | 77.46±1.15 |
| *λ*=0.05 | 75.40±1.58 | 74.60±1.42 | 75.40±1.16 | 77.56±1.40 | 79.73±1.59 | 77.56±1.15 |
| *λ*=0.1 | 63.40±1.73 | 71.00±1.38 | 73.90±1.48 | 74.90±1.44 | 76.96±1.21 | 77.63±1.57 |
| *λ*=1 | 59.60±2.11 | 62.43±1.66 | 54.03±1.33 | 60.20±1.67 | 66.90±1.48 | 70.53±1.56 |
| *λ*=10 | 59.23±1.38 | 50.43±1.54 | 45.66±1.04 | 44.36±1.68 | 41.73±1.50 | 40.66±1.22 |
| *λ*=100 | 58.03±1.35 | 48.90±1.64 | 42.56±1.54 | 43.93±1.26 | 39.03±1.15 | 40.80±0.78 |

**Table S10 The average recognition rates (%) and the corresponding standard deviations (%) of LCJDSRC under different parameters on the validation set of the AR face database with scarf occlusion (sub-image size is 32×32).**

|  | *K*=10 | *K*=20 | *K*=30 | *K*=40 | *K*=50 | *K*=60 |
| --- | --- | --- | --- | --- | --- | --- |
| *λ*=0.0001 | 77.53±1.24 | 79.73±1.09 | 84.23±0.83 | 86.63±1.08 | 86.76±1.11 | 85.96±1.12 |
| *λ*=0.001 | 75.83±1.39 | 83.63±0.61 | 86.76±1.49 | 87.70±0.61 | 86.90±1.04 | 86.56±1.08 |
| *λ*=0.01 | 76.63±0.79 | 81.43±1.37 | 83.10±1.55 | 83.76±1.03 | 83.70±0.90 | 83.30±1.81 |
| *λ*=0.05 | 72.46±1.77 | 76.70±1.39 | 78.43±1.23 | 81.73±1.28 | 81.06±1.88 | 80.10±1.79 |
| *λ*=0.1 | 70.70±2.03 | 74.76±1.64 | 76.66±1.64 | 80.96±1.50 | 80.90±1.17 | 79.36±1.77 |
| *λ*=1 | 60.26±0.73 | 72.83±1.70 | 75.23±1.93 | 79.40±0.82 | 77.70±1.01 | 77.50±1.06 |
| *λ*=10 | 57.10±1.28 | 68.33±1.11 | 73.86±1.57 | 78.96±1.99 | 77.90±1.63 | 76.96±1.11 |
| *λ*=100 | 56.20±0.98 | 67.73±0.96 | 73.16±1.00 | 77.53±1.60 | 76.86±2.00 | 76.83±1.97 |

**Table S11 The average recognition rates (%) and the corresponding standard deviations (%) of LCJDSRC under different parameters on the validation set of the AR face database with sunglasses occlusion (sub-image size is 21×32).**

|  | *K*=10 | *K*=20 | *K*=30 | *K*=40 | *K*=50 | *K*=60 |
| --- | --- | --- | --- | --- | --- | --- |
| *λ*=0.0001 | 82.76±1.57 | 86.46±1.40 | 87.33±1.21 | 88.96±1.02 | 90.20±1.27 | 90.00±1.28 |
| *λ*=0.001 | 84.80±1.64 | 87.96±1.41 | 88.93±0.85 | 90.36±1.02 | 92.83±0.86 | 92.66±0.88 |
| *λ*=0.01 | 83.16±1.40 | 86.86±1.13 | 88.23±1.06 | 89.53±1.09 | 90.00±0.95 | 89.50±0.93 |
| *λ*=0.05 | 81.10±1.52 | 83.76±1.47 | 84.83±1.78 | 88.60±1.36 | 88.26±1.54 | 88.03±1.56 |
| *λ*=0.1 | 81.03±1.93 | 82.96±1.63 | 82.93±1.01 | 85.23±1.90 | 87.70±0.96 | 88.13±1.26 |
| *λ*=1 | 80.53±1.85 | 80.10±1.18 | 82.76±1.58 | 86.93±0.99 | 87.36±1.10 | 88.00±1.82 |
| *λ*=10 | 79.86±1.47 | 81.06±1.45 | 83.76±1.13 | 83.83±1.15 | 87.33±1.15 | 88.90±1.49 |
| *λ*=100 | 78.66±1.07 | 82.30±1.47 | 80.40±1.31 | 84.26±0.64 | 86.63±1.39 | 87.16±1.18 |

**Table S12 The average recognition rates (%) and the corresponding standard deviations (%) of LCJDSRC under different parameters on the validation set of the AR face database with scarf occlusion (sub-image size is 21×32).**

|  | *K*=10 | *K*=20 | *K*=30 | *K*=40 | *K*=50 | *K*=60 |
| --- | --- | --- | --- | --- | --- | --- |
| *λ*=0.0001 | 78.13±1.04 | 83.16±0.54 | 86.80±0.86 | 87.93±1.13 | 85.73±0.89 | 88.20±1.18 |
| *λ*=0.001 | 79.20±1.07 | 87.83±0.90 | 89.50±1.16 | 89.50±1.21 | 91.30±0.85 | 89.53±0.99 |
| *λ*=0.01 | 82.90±1.59 | 89.36±0.82 | 92.66±0.92 | 92.53±0.63 | 92.70±1.03 | 91.36±0.67 |
| *λ*=0.05 | 83.50±0.87 | 90.33±1.01 | 92.93±0.81 | 92.83±0.84 | 93.43±1.03 | 92.80±0.81 |
| *λ*=0.1 | 84.66±0.49 | 89.70±0.79 | 92.70±0.55 | 91.56±0.60 | 93.36±1.02 | 92.83±1.21 |
| *λ*=1 | 78.16±1.50 | 84.80±1.74 | 88.70±1.23 | 89.76±1.37 | 92.63±1.03 | 90.86±1.36 |
| *λ*=10 | 76.33±1.19 | 79.43±1.58 | 83.23±1.33 | 86.20±1.06 | 89.83±1.03 | 89.53±1.16 |
| *λ*=100 | 72.86±1.35 | 78.00±0.92 | 83.33±0.66 | 85.36±0.92 | 87.36±1.10 | 86.46±0.87 |

**Table S13 The average recognition rates (%) and the corresponding standard deviations (%) of LCJDSRC under different parameters on the validation set of the LFW face database (sub-image size is 32×32).**

|  | *K*=10 | *K*=20 | *K*=30 | *K*=40 | *K*=50 | *K*=60 |
| --- | --- | --- | --- | --- | --- | --- |
| *λ*=0.0001 | 38.92±1.12 | 41.89±2.37 | 44.58±1.71 | 46.55±2.32 | 46.36±2.55 | 46.80±2.20 |
| *λ*=0.001 | 39.24±0.58 | 44.24±2.83 | 46.74±2.59 | 47.78±3.09 | 48.03±2.88 | 47.91±2.47 |
| *λ*=0.01 | 40.60±1.22 | 44.14±2.37 | 46.89±2.78 | 46.80±2.17 | 48.63±2.67 | 46.10±2.53 |
| *λ*=0.05 | 41.51±0.49 | 43.25±1.89 | 45.31±2.99 | 45.82±2.98 | 46.96±2.18 | 45.56±1.14 |
| *λ*=0.1 | 39.08±0.87 | 42.72±0.68 | 44.65±2.55 | 45.22±2.61 | 44.14±1.78 | 43.95±1.88 |
| *λ*=1 | 38.95±0.33 | 40.37±1.31 | 42.75±1.78 | 43.70±1.60 | 42.65±1.57 | 41.26±1.87 |
| *λ*=10 | 35.56±0.51 | 39.43±0.38 | 40.56±0.88 | 40.69±0.30 | 39.77±0.30 | 39.58±0.31 |
| *λ*=100 | 35.72±0.11 | 40.82±0.12 | 40.03±2.85 | 40.18±0.33 | 38.95±1.37 | 38.25±0.70 |

**Text S1** The derivation process of Equation (16).

For the *m*th sub-image, Equation (6) can be rewritten as

| $A^{m}=\mathrm{argmin}\vert\vert y^{m}-X^{m}A^{m}\vert\vert_{2}^{2}+\lambda\vert\vert W^{m}\odot A^{m}\vert\vert_{2}^{2}$ | (S1) |
| --- | --- |

Let$S=diag(W^{m})$, through some algebra formulation, Equation (S1) can be rewritten as

|  | (S2) |
| --- | --- |

Through taking partial derivative of Equation (S2) with respect to *A^m^* and setting it to zero, we can obtain the following equation:

|  | (S3) |
| --- | --- |

Finally, we can get *A^m^* as

|  | (S4) |
| --- | --- |
